# High-resolution wind speed forecast system coupling numerical weather prediction and machine learning for agricultural studies — a case study from South Korea

**DOI:** 10.1007/s00484-022-02287-1

**Published:** 2022-04-21

**Authors:** Ju-Young Shin, Byunghoon Min, Kyu Rang Kim

**Affiliations:** High Impact Weather Research Department, National Institute of Meteorological Sciences, 33 Seohobuk-ro, Seogwipo-si, Jeju-do, 63568 South Korea

**Keywords:** Wind speed at 3 m aboveground, Post-processing, Random forests, Machine learning, Downscaling

## Abstract

Forecasting wind speed near the surface with high-spatial resolution is beneficial in agricultural management. There is a discrepancy between the wind speed information required for agricultural management and that produced by weather agencies. To improve crop yield and increase farmers’ incomes, wind speed prediction systems must be developed that are customized for agricultural needs. The current study developed a high-resolution wind speed forecast system for agricultural purposes in South Korea. The system produces a wind speed forecast at 3 m aboveground with 100-m spatial resolution across South Korea. Logarithmic wind profile, power law, random forests, support vector regression, and extreme learning machine were tested as candidate methods for the downscaling wind speed data. The wind speed forecast system developed in this study provides good performance, particularly in inland areas. The machine learning–based methods give the better performance than traditional methods for downscaling wind speed data. Overall, the random forests are considered the best downscaling method in this study. Root mean square error and mean absolute error of wind speed prediction for 48 h using random forests are approximately 0.8 m/s and 0.5 m/s, respectively.

**Supplementary Information:**

The online version contains supplementary material available at 10.1007/s00484-022-02287-1.

## Introduction

Wind speed affects many agricultural activities and crop characteristics, including growth and development (Retuerto and Woodward 2004; Gardiner et al. 2016), and the high value of wind speed can cause fruit falls (McAneney et al. 1984; Gravina et al. 2011), lodging of cereals (Sterling et al. 2003; Feng et al. 2019), and physical damage to crops (Cleugh et al. 1998; Van Gardingen and Grace 1991; Retta et al. 2000). Providing farmers with future wind speed information will increase profits by enabling them to take mitigating actions against adverse impacts of high-speed winds and will improve crop cultivation efficiency (Gardiner et al. 2016). Thus, forecasting wind speed near the land surface is beneficial to agricultural management.

Agricultural production predicted by agricultural simulation model (ASM) using weather forecasts from climate models and numerical weather prediction systems varies with the spatial resolution of weather forecast data (Mearns et al. 1999; Kim et al. 2015). For example, field scale is often preferred for spatial resolution of climate models and numerical weather prediction systems for agricultural use (Hansen and Indeje 2004; Takle et al. 2014; Shin et al. 2020b). For the simulation and forecast of agricultural production, weather prediction data with high spatial resolution should be developed.

To observe and predict wind speed directly affecting agricultural environments, the measuring height of wind speed should be similar to the height of crops. For example, wind speed data at 3 m aboveground are desired for agriculture (Cermak 1979). The standard protocol for measuring wind speed, recommended by WMO (2018), is at a height of wind speed of 10 m aboveground; therefore, climate models and numerical weather prediction system have been designed to simulate and predict wind speed at 10 m aboveground (WS10M). However, due to the difference between anemometer heights and crops, the direct use of wind speed predictions by climate models and numerical weather prediction systems sometimes may be inappropriate in agricultural simulations and modeling for specific purposes such as scheduling pesticide spraying, pollen disposal, lodging prediction, and estimating canopy-top evapotranspiration. Therefore, a methodology is required to downscale wind speed from 10 m aboveground to a lower height aboveground. Wind profiling methods have been developed to predict wind speed at different heights aboveground using WS10M observations, including LOGarithmic wind profile (LOG) and POWer law (POW) methods (Monin and Obukhov 1954; Peterson and Hennessey 1978), and performances and limitations of these methods have been explored in studies (Lubitz 2009; Optis et al. 2016). Recently, machine learning (ML) algorithms have been tested for wind profiling methods (Mohandes et al. 2016; Bodini and Optis 2020; Vassallo et al. 2020); however, most of these studies focused on predicting WS10M; therefore, the utility of these methods for agricultural simulation and modeling is unclear. Moreover, despite its applicability to various fields, climate models and numerical weather prediction systems predict WS10M with a coarse spatial resolution, which causes a discrepancy between the information required by agricultural management and that produced by the weather service. To improve crop yield and increase the income of farmers, wind speed prediction systems that are customized for specific agricultural purposes, need to be developed.

The current study aimed to develop a wind speed forecast system for agricultural purposes where the wind speed data has a 100-m spatial resolution at 3 m aboveground. Korea Meteorological Administration Post Processing (KMAPP) was employed for obtaining WS10M prediction data having 100 m $\times$ 100 m horizontal resolution. For agricultural purposes, wind speeds at 3 m aboveground (WS3M) were measured across South Korea. Furthermore, the WS10M prediction from the KMAPP was downscaled to WS3M using ML algorithms and traditional methods, such as LOG and POW that are constructed by relationship between WS10M predictions and WS3M observations; these downscaled products were compared. Geographical and geological information, as well as other meteorological variables, was tested before using them as input features for the ML methods. The backward elimination method was used to select relevant input features for each ML algorithm for downscaling WS3M.

## High-resolution wind speed forecast system for agriculture

**Fig. 1:**
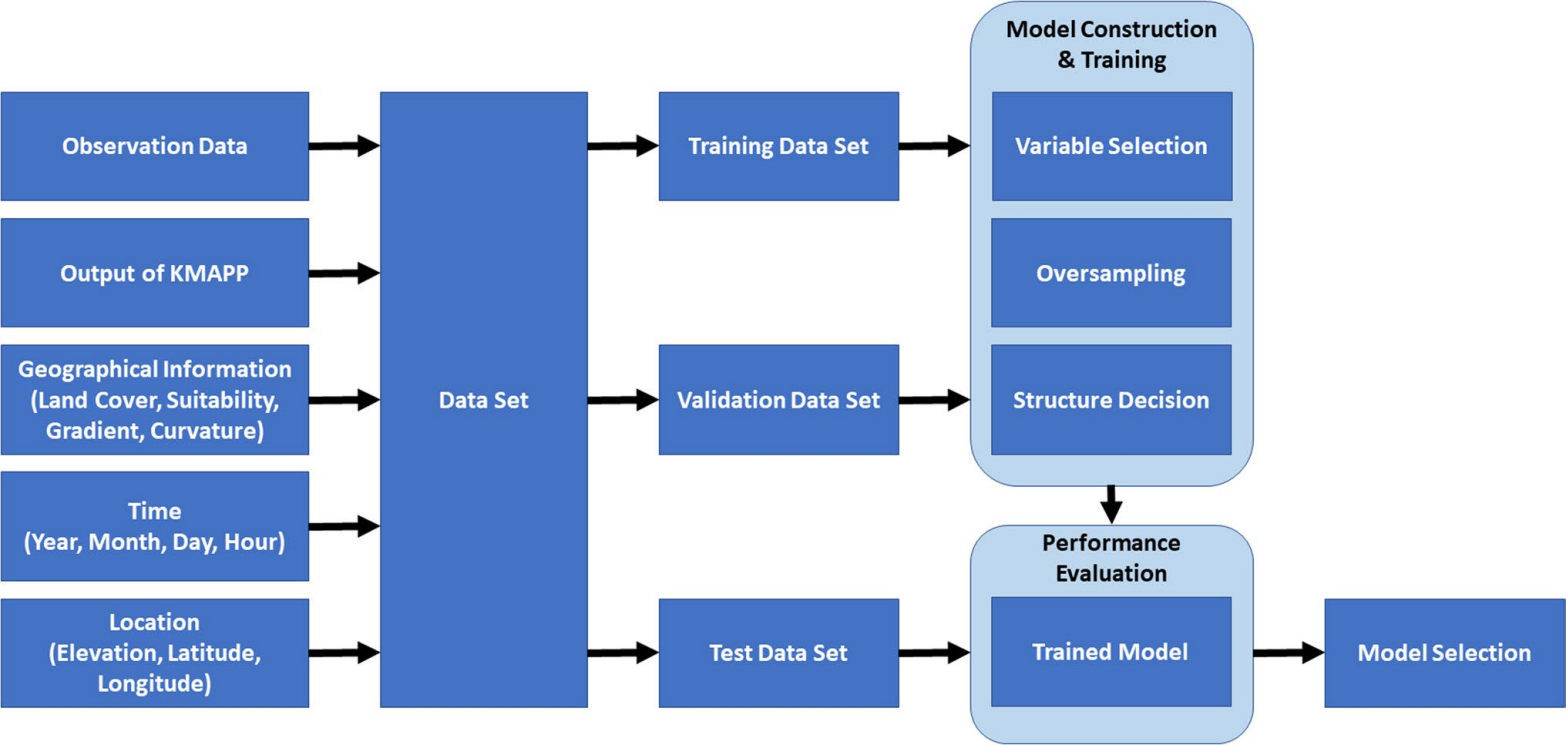
Schematic diagram of the high-resolution wind speed forecast system for agriculture in the current study

In this study, the wind speed prediction system was developed using weather forecast data from numerical weather prediction system and post-processing this data, followed by downscaling wind speed data from 10 to 3 m aboveground. The KMAPP data were used as the weather forecast data. For downscaling methods, several methods including traditional and ML methods are employed. A schematic diagram of the high-resolution wind speed forecast system for agriculture is presented in Fig. 1.

### Weather forecast data from numerical weather prediction system

The Local Data Assimilation and Prediction System (LDAPS) was configured for weather prediction over the Korean peninsula and surrounding waters (KMA 2011). The output of LDAPS has a spatial resolution of 1.5 km (H602 $\times$ V781) and consists of 70 vertical levels up to 40 km (Cho et al. 2020; Kim et al. 2020). Since the spatial resolution is too coarse to be used in other fields, the National Institute of Meteorological Sciences developed KMAPP for producing high-resolution weather forecast data. This model was developed based on the United Kingdom Post Processing method which was proposed by UK met Office. KMAPP produces high-resolution (100 m $\times$ 100 m) weather forecast data using the outputs of LDAPS by spatial downscaling, which is essentially the spatial interpolation of coarse outputs (Yun et al. 2021). Orographic adjustment is adopted for locations with complex terrain. For downscaling wind speed data, additional adjustments are made, based on roughness length and height (Howard and Clark 2007). The reduction rate of air temperature based on height is accounted for while downscaling air temperature (Sheridan et al. 2010, 2018).

KMAPP data consist of surface and level data with an hourly temporal resolution and a lead time of 48 h. Outputs of KMAPP are operationally produced at 00, 06, 12, and 18 UTC within a day. The *u* (east direction) and *v* (north direction) components of WS10M, air temperature, relative humidity, downward shortwave flux, visibility, and mean sea level are produced for surface data in the KMAPP. The level data consists of 29 levels within approximately 3 km that is considered the mixing height. For the level data, *u* and *v* components, air temperature, and air pressure are produced. In the current study, six variables, including *u* and *v* components of WS10M, air temperature, relative humidity, mean sea level pressure, and downward shortwave flux in the surface data of KMAPP, were used as input features for downscaling methods. The information of KMAPP data is summarized in Table S1 in Supplementary Information (SI).

### Downscaling methods

The WS3M for agriculture was predicted by downscaling the WS10M from climate models and numerical weather prediction system. ML algorithms were employed as the primary algorithm for downscaling. However, for comparison, LOG and POW methods were also used as the traditional downscaling method. Random Forests (RF), support vector regression (SVR), and extreme learning machine (ELM) are selected for the candidate of ML algorithm.

#### Logarithmic wind profile method

Based on the LOG method, the mean wind speed at a specific height aboveground (*z* m aboveground) can be calculated by Eq. (1) (Blackadar and Tennekes 1968; Tennekes 1973; Kent et al. 2018):

1 $$\overline{U }\left(z\right)=\frac{{u}_{*}}{\kappa }\mathrm{ln}\left(\frac{z-{z}_{d}}{{z}_{0}}\right)$$

where $\overline{U }\left(z\right)$, ${u}_{*}$, $\kappa$, ${z}_{d}$, and ${z}_{0}$ are mean wind speed at *z* m aboveground, roughness velocity, von Karman’s constant, zero-plane displacement, and roughness length, respectively. The value of $\kappa$ is 0.4, obtained from a wind tunnel experiment (Garratt 1994). WS3M can be predicted by Eq. (2), based on the prediction of WS10M.

2 $$\overline{U }\left(3\right)=\overline{U }\left(10\right)\frac{\mathrm{ln}\left(\frac{3-{z}_{d}}{{z}_{0}}\right)}{\mathrm{ln}\left(\frac{10-{z}_{d}}{{z}_{0}}\right)}$$

#### Power law method

The POW method could describe the relationship between wind speeds at two different heights aboveground. This is defined in Eq. (3) (Emeis 2014; Kent et al. 2018):

3 $$\overline{U }\left({\mathrm{z}}_{1}\right)=\overline{U }\left({\mathrm{z}}_{2}\right){\left(\frac{{z}_{1}-{z}_{d}}{{z}_{2}-{z}_{d}}\right)}^{\alpha }$$

where z_1_, z_2_, and α are wind speed at first height aboveground, wind speed at second height aboveground, and wind shear exponent, respectively. The wind shear exponent ranges from 0 to 1. In this study, Eq. (4) was employed for predicting WS3M based on WS10M using the POW method:

4 $$\overline{U }\left(3\right)=\overline{U }\left(10\right){\left(\frac{3-{z}_{d}}{10-{z}_{d}}\right)}^{\alpha }$$

#### Random forests

RF has been widely used as an alternative for classification and regression problems (Shin et al. 2020a, 2021; Hansen and Indeje 2004; Pal 2005; Smith 2010; Cho et al. 2020; Watt-Meyer et al. 2021). Breiman (2001) proposed the RF algorithm, which uses many decision trees constructed by the bagging method; thus, the RF can be considered as an ensemble of decision trees. Each decision tree is grown using a selected subset that is randomly resampled with randomly selected features from the original datasets. The RF consists of randomness and ensemble learning. The randomness comes from random resampling of the entire dataset and the selection of features with which every classification and regression tree is built. The ensemble learning method in RF means that all individual decision trees in a collection of decision trees (ensemble) contribute to a final prediction. The classification and regression tree, without pruning, is used to construct a single decision tree. The final predicted label is the most frequent among the predicted labels of all individual trees. The “ranger” library in R was used to construct the RF model (Wright and Ziegler 2017).

#### Extreme learning machine

Extreme learning machine is a feed-forward network consisting of a single network with randomly generated weights and bias between input and hidden layers (Huang et al. 2006). The weights and biases of conventional neural network are iteratively optimized while they are tuned in a single iteration because of the randomized weights and biases. The ELM can be expressed in Eq. (5):

5 $$\mathrm{Y}=\mathrm{H\beta }$$

where Y**,** H and β are labels, the output vector of the hidden layer, and weight matrix between hidden layer to output layer, respectively. H is nonlinear feature mapping. H is defined by Eq. (6):

6 $$\mathrm{H}={\mathrm{f}}_{\mathrm{a}}(\mathrm{XW}+\mathrm{B})$$

where ${f}_{a}(\bullet )$, X, W, and B are activation function, input feature, weight matrix between input layer to hidden layer, and bias, respectively. The sigmoid function (${f}_{a}\left(x\right)=\frac{1}{1+\mathrm{exp}(-x)}$) was employed as the activation function in the ELM in this study.

#### Support vector regression

SVR is applied as a regressor to many regression problems in various fields (Yang et al. 2006; Yao et al. 2017; Abbas et al. 2020; Liu et al. 2021; Zhang et al. 2021). The SVR algorithm is a modified version of support vector machine (SVM). The SVR was developed for solving classification problems based on mathematics, unlike other ML algorithms, such as ELM and RF. SVM was developed for building classifiers that maximize the margin, that is, the distance between any two groups. The distance between two groups is determined by the distance between support vectors, that is, the nearest vector to another group. Unlike the SVM, the support vectors indicate the two most distant vectors in the datasets for the SVR. Additionally, the regression line that minimizes the margin represents the regressor in the SVR. To figure out the hyperplane minimizing the margin, the optimization problem (Eqs. (7)–(9)) with its constraints has to be solved:

7 $$\mathrm{min}\tau \left(\mathrm{W},\xi ,\rho \right)=\frac{1}{2}{\Vert \mathrm{W}\Vert }^{2}-\nu \rho +\frac{1}{2}{\sum }_{i=1}^{n}{\xi }_{i}$$

subject to $${\mathrm{y}}_{\mathrm{i}}\left(\Theta \left({\mathbf{X}}_{\mathbf{i}}\right)\mathbf{W}+b\right)\le \rho -{\xi }_{i}, i=1,\dots ,n$$ (8)

9 $${\upxi }_{\mathrm{i}}\ge 0,\uprho \ge 0$$

where, v ${\xi }_{i}$, and ${\varvec{\Theta}}\left(\bullet \right)$ are regularization constant that ranges from 0 to 1, slack variable for *i*th data point, and kernel function. The radial basis function is used for kernel function in the SVR used in this study, and the “e1071” library in the R was employed for conducting SVR (Meyer et al. 2021).

## Data

### Meteorological data

**Fig. 2:**
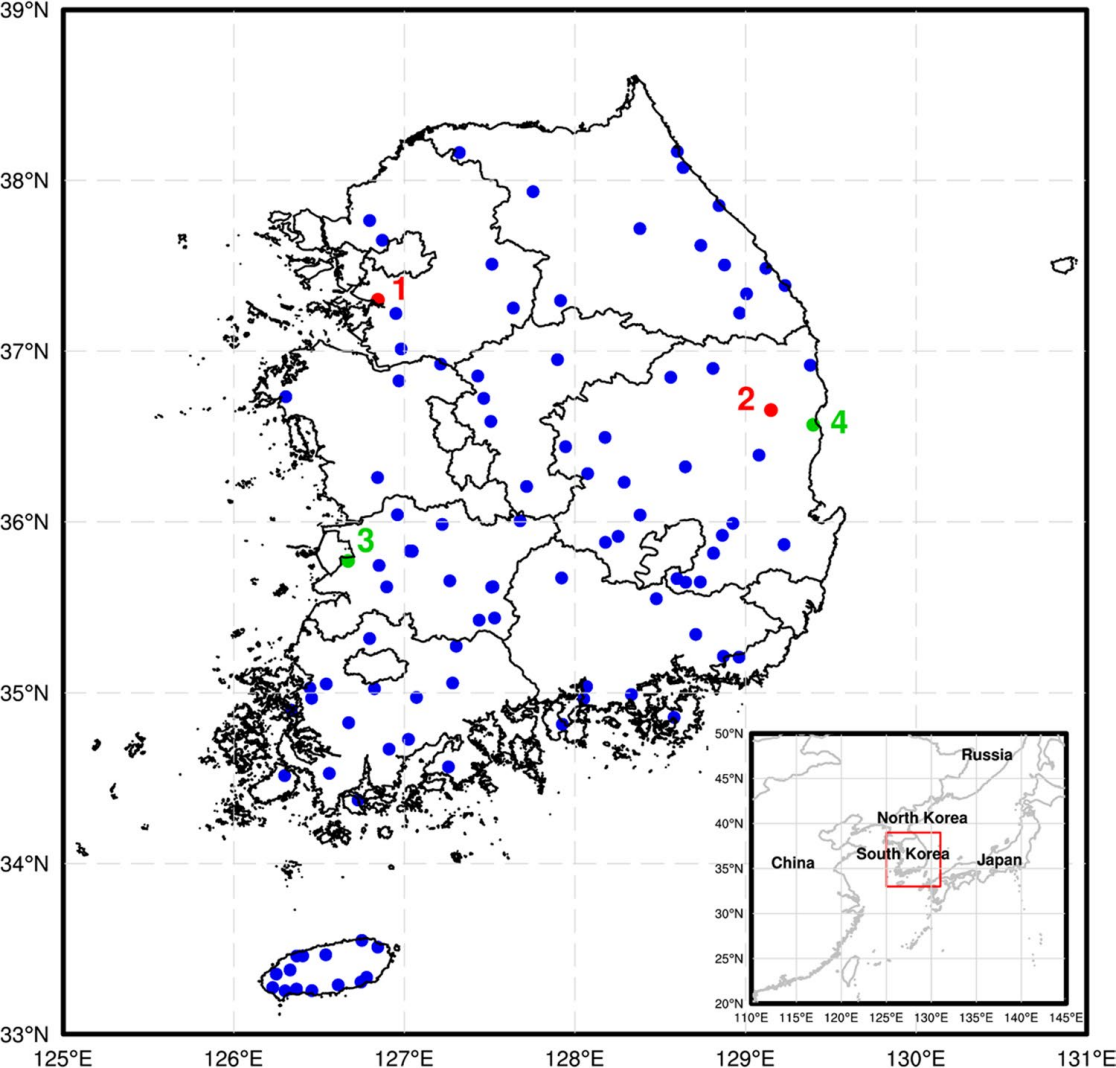
Location of the used weather stations. Note that the red and green colored stations are selected for performance evaluation at individual stations while all stations are employed for evaluating overall performances of the developed model

In this study, meteorological data from the Automated Agricultural Observing System (AAOS) were used. AAOS stations are located in agricultural areas, such as farms and orchards, and measure 14 meteorological variables related to the agricultural environment, e.g., air temperature, wind speed, and relative humidity. In this study, hourly observed WS3M data from AAOS were employed for the target data. Hourly wind speed depicts 10-min mean wind speed data observed at 00 min in each hour. The wind speed data can be downloaded from Nongeupnalssi 365 (weather.rda.go.kr). The recording period was from July 2019 to June 2020 (12 months). To obtain reliable data, the meteorological observation data were inspected by a quality check (QC) procedure based on KMA guideline of QC for automatic weather station, using the relocation of the measuring instrument, location of the measuring instrument, proportion of missing data, and proportion of zero values. The stations where the displacement of instrument is higher than 150 m were extracted; in addition, when the instruments were installed in improper location such as near road and high building, the data in these stations were not used. If proportions of missing data and null wind speed were higher than 10%, measures in these stations were not employed. Based on the QC results, 104 stations were of sufficient quality to be used in the modeling. The selected 104 stations are presented in Fig. 2.

### Terrain-based spatial data

Surface wind speed varies with surface characteristics such as topography and land cover (Wu et al. 2016). Use of these surface characteristics for input features of machine learning algorithm led to improvements in near-surface wind speed prediction (Jung and Schindler 2020). In this study, land cover and agricultural suitability maps were used as input features for the ML methods as they are related to the agricultural environment. Land cover maps represent the land cover type at the specific location, based on predefined categories, and have three levels: top, medium, and low, based on the hierarchy of land cover types. These types in the map provide the most detailed information at the low level. Medium-level map, with a scale of 1:25,000 that horizontal resolution is approximately less than 10 m, was used in this study as it has sufficient detail to represent land cover characteristics in agricultural areas. This data was obtained from the environmental geographic information service (egis.me.go.kr). The agricultural suitability map, with a scale of 1:25,000 scale, includes information related to cultivation, e.g., soil properties, yield potential, and degree of yield constraint, to help decision-making for increasing agricultural production and yield. The agricultural suitability map used in this study can be downloaded from the Korean soil information system (soil.rda.go.kr). The land cover and agricultural suitability maps are transformed to grid data that matches the KMAPP grid, using the nearest neighbor resampling method.

Terrain is critical in determining wind speed, because air flow is largely associated with terrain, such as mountains, valleys, and flat planes (Cao and Tamura 2006; Schmidli and Rotunno 2012). Elevation data at a specific location may have a limited capacity to represent topological characteristics; hence, the slope and curvature were used as input features representing topological characteristics in this study. The slope and curvature were calculated from the digital elevation map used in KMAPP. These calculated slope and curvature were then employed as input features of the ML algorithm. The slope indicates a first-order differential elevation, with respect to location, and has been widely used in geomorphometrics (Evans 1972; Ghandehari et al. 2019). The curvature is defined as a second-order differential elevation, with respect to location (Ghandehari et al. 2019).

## Application

Traditional methods estimate WS3M using WS10M predictions from KMAPP. To compute WS3M using traditional methods, some parameters, such as ${z}_{0}$, $\alpha$, and ${z}_{d}$ should be defined. In this study, ${z}_{0}$ and $\alpha$ were given based on the land cover type of the location (Aghbalou et al. 2018; Chavan et al. 2017). The values of ${z}_{0}$ and $\alpha$ used in this study are listed in Tables S2 and S3 in SI. The value of ${z}_{d}$ was set to 2/3 of the canopy height, assuming that the canopy height is 1 m (De Bruin and Verhoef 1997).

For WS3M prediction using ML algorithms, 801,769 data points were collected. Sixty (481,051), 20 (160,359), and 20% (160,359) of the all data points used were randomly resampled for training, validation, and test datasets, respectively, without replacement. Thus, the number of data points for the validation and test datasets was 160,359 and 160,359, respectively. The observed WS3M is unbalanced data in that the values of data are not uniformly distributed. The use of unbalance data leads to overfitting of model for the most frequent values. Thus, this unbalance data can worsen performances for less frequent values. To avoid overfitting from the unbalanced data, oversampling was performed for the training dataset. In oversampling, the number of data point in each bin that has 1 m/s interval becomes the same by resampling data points in the training dataset. Hence, the number of data points for the training data set was 16,113,150. To optimize input feature selection, backward elimination was carried out (Xu and Zhang 2001). Forty-eight features were considered as the initial input feature in the backward elimination method, including six meteorological variables predicted by the KMAPP, 30 features from the agricultural suitability map, one feature from the land cover map, two features from the slope, two features from the curvatures, four features indicating time, and three features indicating geological location. The input features of all ML methods were selected using the backward elimination method, based on root mean square error (RMSE) for the validation dataset. According to the backward elimination method, the ML methods employed a total of 18 input features: six from KMAPP (*u* and *v* components of WS3M, air temperature, relative humidity, mean sea level pressure, and downward shortwave flux), three from the agricultural suitability maps (drainage level, land form, and soil suborder), one from the land cover map, two from the slopes (of north and east direction), two from the curvatures (of north and east direction), one feature from time (month), and three features from geological information (elevation, latitude, and longitude).

The hyperparameters of the ML methods were optimized based on the RMSE value of the trained models for the validation dataset. To determine the number of trees in RF, 300–700 trees were tested. When there were 500 trees, the RMSE of the trained RF was the lowest; hence, 500 trees were used in the RF. For the SVR, 0.01–1 were tested for gamma, and 1–10 were tested for the cost using validation dataset. Based on the cross validation, the smallest RMSE was obtained when the gamma and cost were equal to 0.05 and 1, respectively. The hyperparameters of ELM used in this study were the number of nodes and the tuning parameter. For ELM, 1000–4000 nodes were tested and 0.01–10 were tested for the tuning parameter, and 2000 and 0.1 were used as the number of nodes and tuning parameter, respectively, based on the cross validation.

## Results

### Overall performance of models

**Fig. 3:**
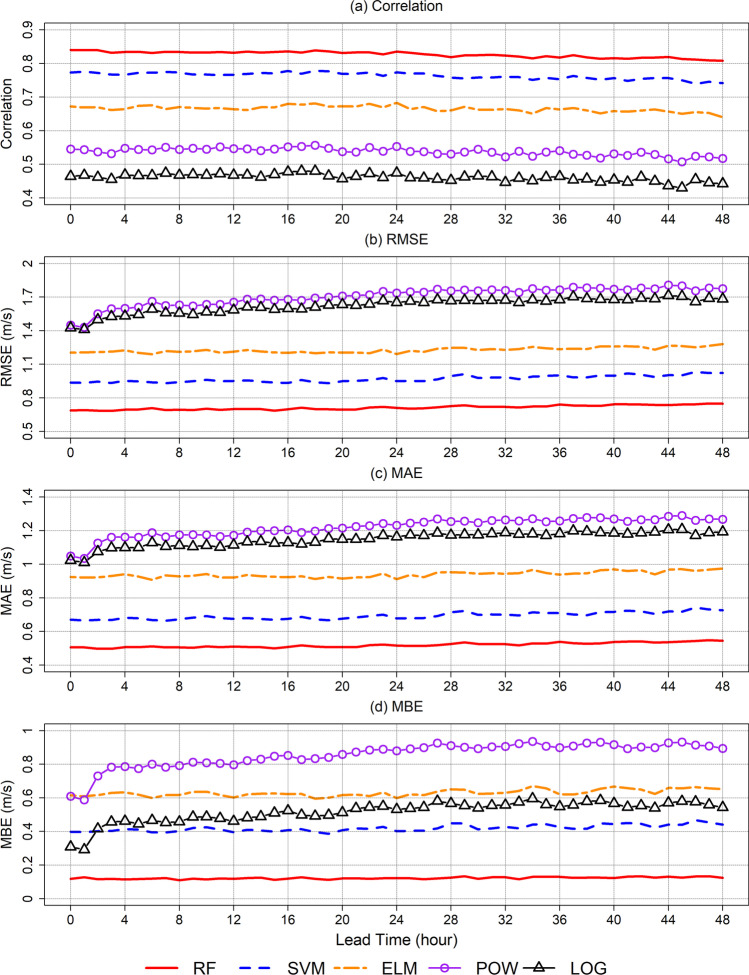
Performance evaluation of five models for all stations using the test data set based on four evaluation measures: **a** correlation, **b** RMSE, **c** MAE, and **d** MBE

All evaluation measures were calculated using the test dataset. The proposed system predicts WS3M at 4.6 million points across South Korea. WS3M prediction at 104 points that match the AAOS weather stations were used for a performance evaluation. Correlation, RMSE, mean absolute error (MAE), and mean bias error (MBE) of WS3M predictions from all the models are presented in Fig. 3. Based on correlation, RMSE, and MAE, the ML-based methods performed better than LOG and POW methods. Among the ML algorithms, RF was the best method, while ELM performed the worst. The RMSE value of the prediction for RF was < 0.8 m/s, while that for ELM was > 1.1 m/s. The RMSE values for traditional wind profiling methods, LOG and POW, were > 1.4 m/s. Similarly, RF had the best performance for all lead time with MBE < 0.2 m/s. The MBE of ELM and LOG was > 0.6 m/s. Among the methods, POW had the largest MBE value > 0.9 m/s with a long lead time. Prediction performances of ML-based methods gradually decreased as lead time increased, whereas the prediction performances of traditional methods drastically reduced after a 2-h lead time. The mean, median, and standard deviation of observed WS3M for all employed stations are presented in Figure S1. The mean value of mean and median wind speed is 1.27 m/s and 0.98 m/s, respectively. Thus, the approximate relative absolute error ($=\frac{0.5}{1.27}\times 100$) based on mean value of MAE (0.5 m/s) for WS3M prediction by RF is 39.4%.

**Fig. 4:**
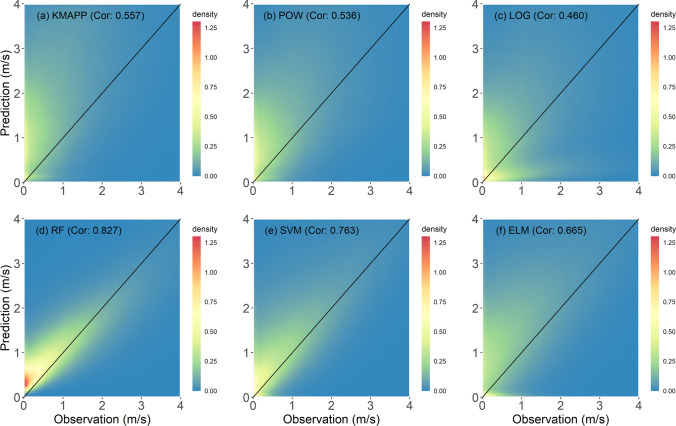
Density plots of five models as well as KMAPP for all stations using the test data set: **a** KMAPP, **b** POW, **c** LOG, **d** RF, **e** SVR, and **f** ELM

**Fig. 5:**
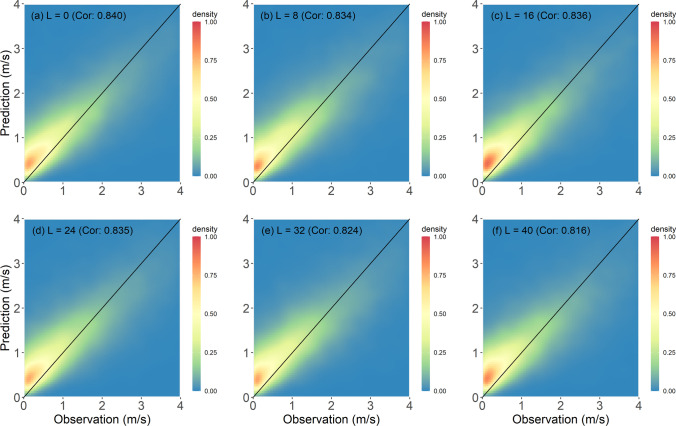
Density plots of prediction from the RF model for different lead times using the test data set: **a** lead time (*L*) = 0 h, **b** *L* = 8 h, **c** *L* = 16 h, **d** *L* = 24 h, **e** *L* = 32 h, and **f** *L* = 40 h

Figure 4 represents the overall performances of the tested models for all stations evaluated using density plots comparing WS3M and the KMAPP prediction for WS10M. The predictions of KMAPP are higher than the observed wind speed due to the difference in measuring height resulting in a correlation value of 0.557. Though the LOG and POW provided lower wind speed than the KMAPP, large positive biases remain in their predictions. The correlations for POW and LOG were 0.536 and 0.460, respectively. The predictions of RF and SVR were found to have good agreement with the observations, as their scatter points are located around the diagonal line. Unlike RF and SVR, the ELM led to an overestimation. The RF method showed the highest correlation with a value of 0.827. However, the RF algorithm overestimated wind speed lower than 1.5 m/s and underestimated above 2 m/s. The SVR had the second-highest correlation with a value of 0.763. Overall, the SVR overestimates wind speed. Based on the evaluation measures, RF was determined as the best method; hence, the prediction performance of RF for various lead times was described using the density plot. These density plots for predictions at nowcasting (0 h), 8, 16, 24, 32, and 40 h are presented in Fig. 5. The correlations decrease as lead time increases, and the distributions of scatter points for different lead times were found to be similar. The highest correlation (0.84) was found for predictions at current time.

### Spatial distribution of evaluation measures

**Fig. 6:**
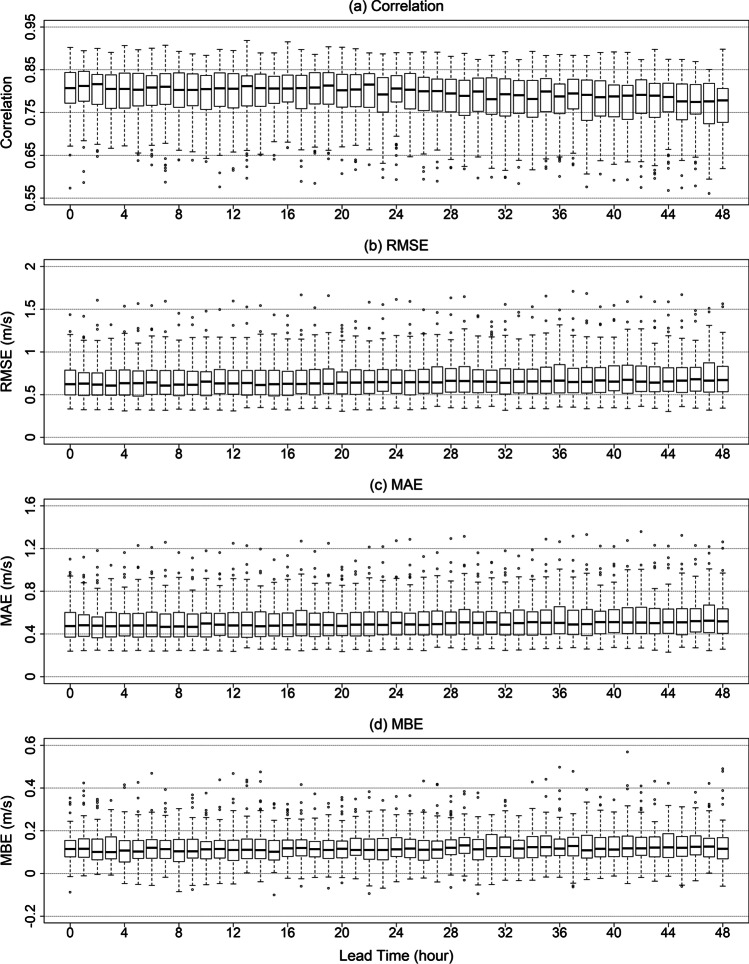
Boxplots of the employed evaluation measures for all lead time in all stations: **a** correlation, **b** RMSE, **c** MAE, and **d** MBE

To further investigate the prediction performance of the RF algorithm, correlation, RMSE, MAE, and MBE for each lead time and station are presented in Fig. 6. The medians of correlations were approximately 0.8, which decrease as the lead time increases. Spatial variation of evaluation measures (depicted as ranges of boxes) ranged from 0.7 to 0.85 and was consistent for all lead times. The results indicate that the spatial variation of prediction performances for all models is consistent for all lead times. Overall tendencies of RMSE, MAE, and MBE are similar to the correlation. For MAE, the medians are approximately 0.5 m/s, and its spatial variation ranged from 0.3 to 0.7 m/s. Medians of RMSE were between 0.6 and 0.7 m/s, and the its spatial variation ranged from 0.5 to 0.9 m/s. For MBE, the medians were approximately 0.1 m/s, and its spatial variation was between 0 and 0.2 m/s.

**Fig. 7:**
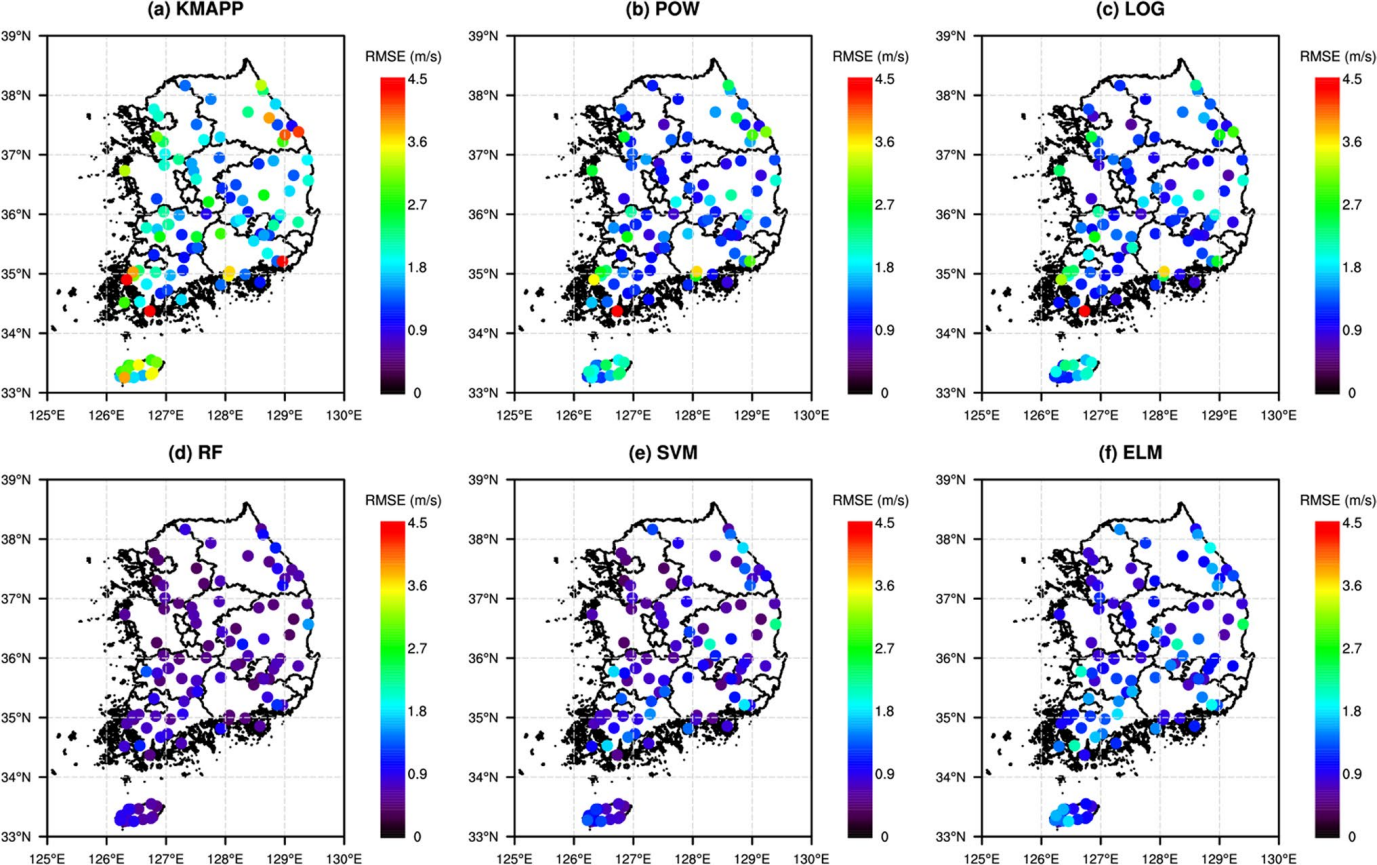
Spatial distributions of RMSE for six models in the used stations: **a** KMAPP, **b** POW, **c** LOG, **d** RF, **e** SVR, and **f** ELM

**Fig. 8:**
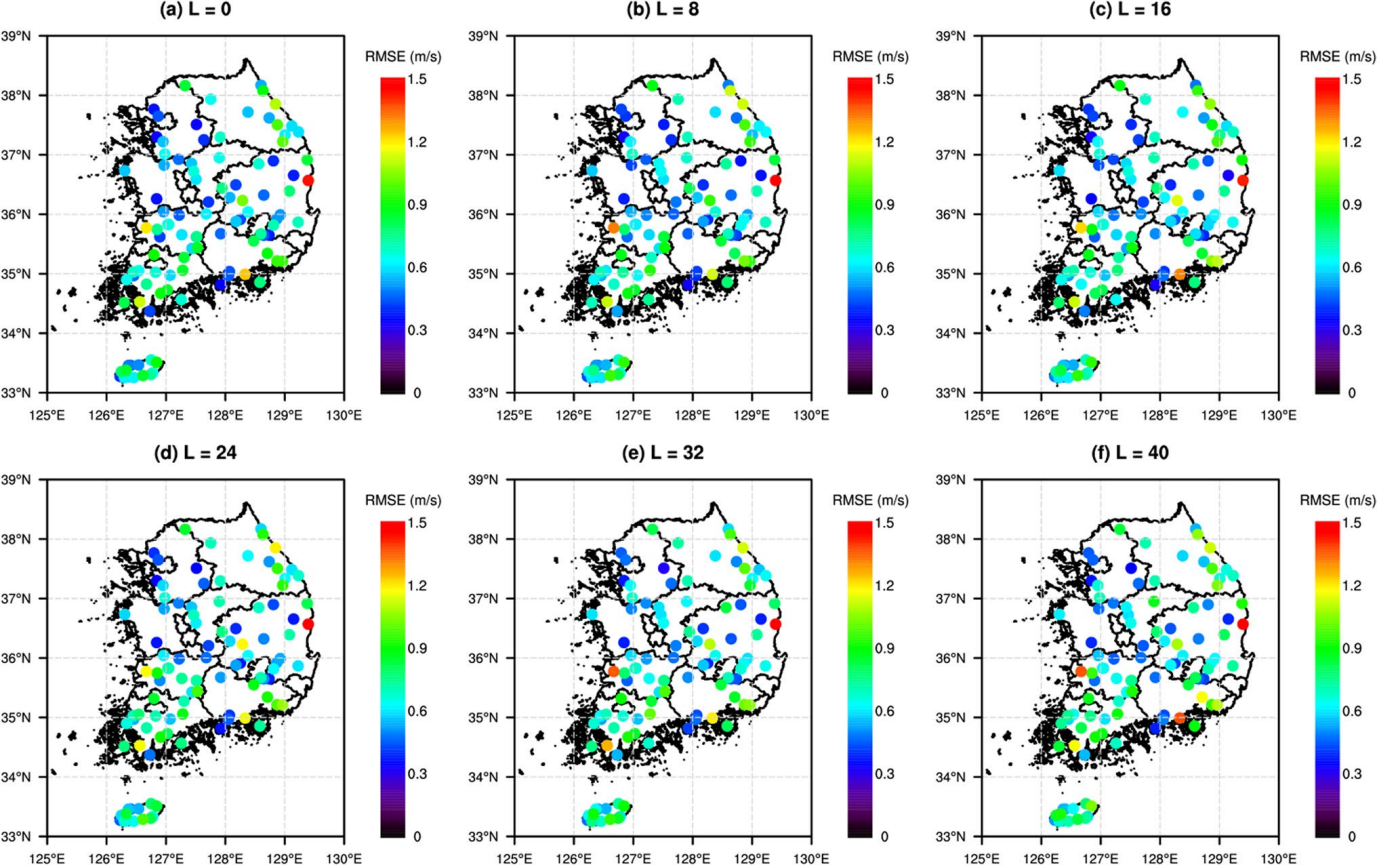
Spatial distributions of RMSE from RF for different lead time (L) in the used stations

The spatial distribution of RMSE from POW, LOG, RF, SVR, and ELM is presented in Fig. 7, to investigate the spatial characteristics of prediction performances for all models. The spatial distribution of RMSE for KMAPP is also illustrated for comparison. For all models, RMSEs for stations in coastal region are larger than in inland regions. The spatial distribution of RMSEs for KMAPP was also similar. The RMSEs for stations in inland regions are > 1.8 m/s, while the RMSEs for stations in coastal regions are > 3.6 m/s. Traditional methods, such as POW and LOG, lead to smaller RMSE than KMAPP, but RMSEs for some coastal stations are > 3.6 m/s, even though the wind speed is downscaled at 3 m aboveground. The ML-based models provide smaller RMSEs than the traditional methods; the RF gives the smallest RMSEs of all the models (< 1.8 m/s for all stations). To investigate the variation of performance depending on lead time, the RMSEs for RF in six lead-times are presented in Fig. 8. For six lead times, the RMSEs for stations in coastal regions were larger than in inland regions. RMSEs increase as the lead time becomes longer. The increments of RMSEs for stations in coastal region are larger than in inland regions.

## Discussion

In this study, traditional and ML methods were employed for downscaling wind speed data and predicting WS3M. Overall, the ML methods lead to a better prediction performance than traditional methods. The poor performances of traditional methods for downscaling may result from the use of inaccurate values for ${z}_{0}$, $\alpha$, and ${z}_{d}$. The values of these parameters can be accurately obtained by considering complex conditions, such as type, density, and height of vegetation. Some studies have reported that these parameters are associated with the type and density of vegetation (Shaw and Pereira 1982); therefore, information on these parameters is needed to successfully implement traditional methods for downscaling wind speed data in agricultural areas. The crop growth stage depends on various conditions, such as weather, vegetation type, soil properties, farmers, and days after sowing. The crop growth stage can represent the height and density of vegetation; thus, these parameters are strongly linked to the crop growth stage. However, collecting all the information related to changes in the crop growth stage for farm areas nationwide is virtually impossible. Thus, in traditional methods, the inaccurate estimations of these parameters are an inevitable limitation. Overcoming some of the limitations of traditional methods, the ML methods provide a better prediction performance for downscaling wind speed in agricultural areas. ML algorithms can incorporate detailed information of surface, meteorology and meteorological prediction, and time. Although these parameters cannot directly represent vegetation properties in the area of interest, they can indirectly represent vegetation, and thus, improving the performance of the ML method in predicting WS3M.

Among the tested ML methods, RF performed the best at downscaling the wind speed data. The ELM algorithm performed the worst, but its performance was better than traditional methods. Agricultural suitability map and land cover map (categorical variables) were used as input features, e.g., mountain, flat plane, and slope near mountain. The ELM method may have poor performance because of the activation functions, e.g., sigmoid and hyperbolic tangent functions, which are normally used for continuous numeric variables. The categorical variable such as the farm for land cover cannot be directly used in the ELM, and this variable has to be digitized like one or two for use in the ELM. The ELM inherently accounts for the magnitude of digits for input feature as strength of signal due to the activation function. However, because the magnitude of digits for the digitized categorical variable is meaningless, the ELM may not fully use information from the categorical variable.

Performance of wind speed prediction in inland areas is better than that in coastal areas for all tested downscaling methods. The performance difference between two areas results from wind speed prediction skill of KMAPP. The KMAPP provides better prediction skill for WS10M in inland areas than that in coastal regions (Yun et al. 2021). Because the WS3M prediction comes from the WS10M prediction by KMAPP, the prediction performance of WS3M is strongly associated to WS10M prediction. The result supports that the difference comes from the difference between prediction skills in inland and coastal areas. Subsequently, the proposed WS3M prediction system provides better performance in inland region as compared to the performance in coastal areas.

For some agricultural purposes, wind speed prediction at lower height than 10 m is required. WS3M data is valuable information in modeling canopy-top evapotranspiration (Allen et al. 1998). Additionally, when unmanned aerial vehicles (UAVs) are used to spraying pesticide, the flying height should be lower than 4 m in South Korea due to the government regulation on avoiding long drift of pesticide (NAAS 2018). Though there is no standard flying height for UAVs, the flying height is less than 5 m aboveground (Martin et al. 2019). Thus, WS3M can be used to make decision to spray pesticides onto farm using the UAV in South Korea. For modeling serial crop lodging, wind speed at 2 m aboveground often has been adopted in some studies (Wen et al. 2019). In addition, the information of wind speed at lower height than 10 m can be used to model pollen disposal, which can be used in yield modeling (Tackenberg 2003). The height of wind speed data should be selected based on type of plants. Hence, there is no consensus for use of WS3M in agricultural modeling and simulation. For instances, the American Society of Agricultural and Biological Engineers recommended wind speed data at from 2 to 3 m (ASBAE 2006), the American Association of State Climatologists recommended WS3M (AASC 1985), and WMO recommended 2 m for height of anemometer (Gommes et al. 2010). In downscaling wind speed at low height that is lower than 3 m, use of WS3M may lead to more accurate data than use of WS10M. However, it needs to explore what is an appropriate height for wind speed measure in agriculture.

The downscaling methods proposed in this study have certain limitations. First, the downscaling methods provide poor performances for downscaling WS3M near the coast. The RMSE values of stations in inland areas are < 1 m/s, and it varies depending on whether lead time is small. However, the RMSEs of stations near the coast are larger than in inland areas, and the variation of RMSE depending on lead time is large. Hence, further research is required to improve the performance of the downscaling method for wind speed near the coast. Second, the proposed downscaling system provides an inaccurate WS3M prediction for wind speed greater than 4.5 m/s. The RF methods led to an underestimation of high value of wind speed. Hence, the developed models have a limited capacity for warning the risk or damage from high winds. High values of wind speed near surface are strongly associated to the turbulent kinematic energy under mixing height (Seibert et al. 2000; Brasseur 2001). In this study, because near-surface wind speed predictions from numerical weather prediction model were used for predicting WS3M, turbulent kinematic energy was not considered. This limitation may lead to poor performance for predicting high wind speed. Thus, to improve predictability of high wind speed, the prediction data for high altitudes will be considered in wind speed prediction. Third, the proposed ML-based models can only predict WS3M. The ML-based models were trained using WS3M; therefore, these models cannot predict wind speed at different heights. Although the traditional methods can predict wind speed at various heights, a large amount of information is required to implement these methods accurately. Thus, these limitations should be explored in future research. Also, based on results of backward elimination method, slope and curvature representing characteristics of terrain were selected for input variables. The characteristics of terrain would be associated to wind speed mechanism at low altitude. In this study, quantifying how terrain is relevant in WS3M prediction was not carried out for consistency. Investigating impact of terrain on wind speed at low altitude is a good research question to improve our understanding on wind speed mechanism, particularly in agriculture areas. Hence, a relationship between terrain and wind speed, particularly in agriculture areas, should be investigated in future research.

## Conclusions

The current study developed a high-resolution wind speed prediction system for agriculture purposes. The developed system consists of two parts: WS10M prediction and downscaling WS10M to WS3M. For downscaling method, traditional and ML-based methods were employed. The performance of WS3M prediction from the developed system was evaluated using the observed WS3M across South Korea. The developed wind speed prediction system provides a good performance for predicting WS3M, and this can be beneficial for agricultural applications. This performance can provide valuable wind speed information for scheduling pesticide spraying, pollen disposal, lodging prediction, and estimating canopy-top evapotranspiration in South Korea. The ML-based methods are more appropriate to predict wind speed at a fixed height, e.g., 3 m, than the traditional methods. Because the ML-based methods can integrate various variables related to wind speed and the developed model inherently pursue predicting wind speed at a fixed height, they can lead to better performances for predicting wind speed at a fixed height than the traditional methods which use roughness length, zero-plane displacement, and wind shear exponent. RF is considered as the most appropriate algorithm of the tested ML algorithms for downscaling wind speed to the fixed height. The ML method, e.g., ELM, which has the limited capacity to consider categorical variable may need delicate procedure to preprocessing the categorical variables for considering them. Thus, applying an algorithm that is easy to consider categorical variables would lead to successful implementation for wind speed prediction. These results bolster the fact that the ML algorithm, which can successfully consider categorical variables, would be a good option for predicting weather variables related to agriculture.

## Supplementary Information

Below is the link to the electronic supplementary material.

Supplementary file1 (DOCX 977 KB)

## Data Availability

Meteorological data can be downloaded from weather.rda.go.kr, and the spatial data can be downloaded from egis.me.go.kr and soil.rda.go.kr. Outputs of KMAPP can be obtained by requesting the data to the National Institute of Meteorological Sciences (www.nims.go.kr).
