# Supplementary material for: High-resolution wind speed forecast system coupling numerical weather prediction and machine learning for agricultural studies — a case study from South Korea
Source: Int J Biometeorol. 2022 Apr 21;66(7):1429–43. doi: 10.1007/s00484-022-02287-1 (PMC9151559; doi:10.1007/s00484-022-02287-1)
Supplement: Supplementary file 1 — Supplementary file1 (DOCX 977 KB) [file 484_2022_2287_MOESM1_ESM.docx]

# **Supplementary information of “****High-resolution wind speed forecast system coupling numerical weather prediction and machine learning for agricultural studies— A case study from South Korea”**

Ju-Young Shin, Byunghoon Min, and Kyu Rang Kim

Table S1 Details of the Korea Meteorological Administration Post Processing (KMAPP) data

| No. | Element | Height (m) | Unit |
| --- | --- | --- | --- |
| 1 | Air temperature | 1.5 | ℃ |
| 2 | Relative humidity | 1.5 | %/100 |
| 3 | U component | 10 | m/s |
| 4 | V component | 10 | m/s |
| 5 | Downward shortwave flux | - | W/m^2^ |
| 6 | Visibility | 1.5 | m |
| 7 | Mean sea level pressure | - | mb |

Table S2 Typical value of surface roughness length $\boldsymbol{z}_{\boldsymbol{0}}$ and classification code of land cover

| Type | $\boldsymbol{z}_{\boldsymbol{0}}$ | [Aghbalou et al. (2018](#_ENREF_1)) | [Chavan et al. (2017](#_ENREF_2)) |
| --- | --- | --- | --- |
| Water bodies | 0.0002 | 0.0002 | 0.0002 ~ 0.0003 |
| Bare land | 0.001 | - | 0.001 |
| Grassland | 0.01 | 0.01 | 0.01 ~ 0.05 |
| Cropland | 0.05 | 0.05 | - |
| Orchard | 0.1 | 0.1 | - |
| Forest | 0.5 | 0.5 | 0.1 ~ 1 |
| Suburbs | 1.5 | 1.5 | - |
| Centers of cities | 3.0 | 3.0 | 1 ~ 4 |

Table S3 Typical value of wind shear exponent $\boldsymbol{\alpha}$ and classification code of land cover

| Type | $\boldsymbol{\alpha}$ | [Aghbalou et al. (2018](#_ENREF_1)) | [Chavan et al. (2017](#_ENREF_2)) |
| --- | --- | --- | --- |
| Water bodies | 0.1 | 0.1 | 0.1 |
| Bare land | 0.1 | 0.1 | 0.1 |
| Grassland | 0.13 | - | 0.13 ~ 0.135 |
| Cropland | 0.18 | 0.2 | 0.128 ~ 0.170 |
| Orchard | 0.21 | 0.2 | 0.22 |
| Forest | 0.24 | 0.25 | 0.23 |
| Suburbs | 0.3 | 0.3 | 0.28 ~ 0.30 |
| Centers of cities | 0.4 | 0.4 | 0.4 |


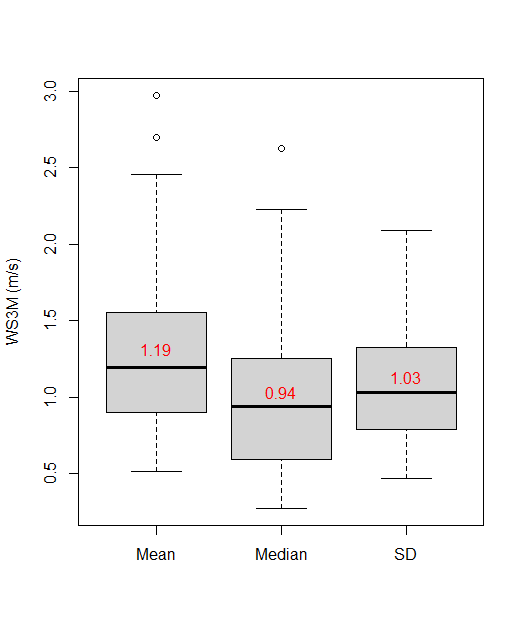


Figure S1 Mean, median and Standard Deviation (SD) distribution of observed WS3M for employed 104 stations.

**References**

Aghbalou N, Charki A, Elazzouzi SR, Reklaoui K (2018) A probabilistic assessment approach for wind turbine-site matching. In J Elec Power & Energy Syst 103:497-510. doi:10.1016/j.ijepes.2018.06.018

Chavan DS, Gaikwad S, Singh A, Parashar D, Saahil V, Sankpal J, Karandikar P Impact of vertical wind shear on wind turbine performance. In: 2017 International Conference on Circuit, Power and Computing Technologies (ICCPCT), 2017. IEEE, pp 1-6. doi:10.1109/ICCPCT.2017.8074395
